# Punicalagin Induces Nrf2/HO-1 Expression via Upregulation of PI3K/AKT Pathway and Inhibits LPS-Induced Oxidative Stress in RAW264.7 Macrophages

**DOI:** 10.1155/2015/380218

**Published:** 2015-04-19

**Authors:** Xiaolong Xu, Hongquan Li, Xiaolin Hou, Deyin Li, Shasha He, Changrong Wan, Peng Yin, Mingjiang Liu, Fenghua Liu, Jianqin Xu

**Affiliations:** ^1^CAU-BUA TCVM Teaching and Researching Team, College of Veterinary Medicine, China Agricultural University (CAU), No. 2 West Yuanmingyuan Road, Beijing 100193, China; ^2^College of Animal Science and Veterinary Medicine, Shanxi Agricultural University, Taigu, Shanxi 030801, China; ^3^College of Animal Science and Technology, Beijing University of Agriculture (BUA), Beijing 102206, China; ^4^Beijing Key Laboratory for Dairy Cow Nutrition, Beijing 102206, China

## Abstract

Reactive oxygen species (ROS) and oxidative stress are thought to play a central role in potentiating macrophage activation, causing excessive inflammation, tissue damage, and sepsis. Recently, we have shown that punicalagin (PUN) exhibits anti-inflammatory activity in LPS-stimulated macrophages. However, the potential antioxidant effects of PUN in macrophages remain unclear. Revealing these effects will help understand the mechanism underlying its ability to inhibit excessive macrophage activation. Hemeoxygenase-1 (HO-1) exhibits antioxidant activity in macrophages. Therefore, we hypothesized that HO-1 is a potential target of PUN and tried to reveal its antioxidant mechanism. Here, PUN treatment increased HO-1 expression together with its upstream mediator nuclear factor-erythroid 2 p45-related factor 2 (Nrf2). However, specific inhibition of Nrf2 by brusatol (a specific Nrf2 inhibitor) dramatically blocked PUN-induced HO-1 expression. Previous research has demonstrated that the PI3K/Akt pathway plays a critical role in modulating Nrf2/HO-1 protein expression as an upstream signaling molecule. Here, LY294002, a specific PI3K/Akt inhibitor, suppressed PUN-induced HO-1 expression and led to ROS accumulation in macrophages. Furthermore, PUN inhibited LPS-induced oxidative stress in macrophages by reducing ROS and NO generation and increasing *superoxide dismutase (SOD) 1* mRNA expression. These findings provide new perspectives for novel therapeutic approaches using antioxidant medicines and compounds against oxidative stress and excessive inflammatory diseases including tissue damage, sepsis, and endotoxemic shock.

## 1. Introduction

Reactive oxygen species (ROS) play a vital role in LPS-triggered macrophage activation by regulating intracellular reduction-oxidation (redox) sensitive signaling pathways and nuclear transcription factors, such as nuclear factor-*κ*B (NF-*κ*B) and nuclear factor-erythroid 2 p45-related factor 2 (Nrf2) [1–3]. Overproduction of ROS in macrophages leads to excessive expression of cytokines and inflammatory factors, resulting in atheromatous plaques, acute inflammation, tissue injury, and sepsis [4]. However, most cell types have developed defensive mechanisms to counteract ROS generation [5, 6]. Hemeoxygenase-1 (HO-1), a member of the intracellular phase II enzyme family, is thought to play an essential role in maintaining cellular redox homeostasis against ROS generation and oxidative stress [7, 8]. HO-1, which is expressed in cells at a low level without stimulation, can be rapidly induced by various oxidative-inducing agents, including LPS [9], heme [10], hypoxia, and subsequent lethality [11]. In oxidative stress and inflammation conditions, enhancement of HO-1 expression plays an important role in cell protection [9, 11]. This important cytoprotective action in response to various cellular stimuli makes it conceivable to target HO-1 induction as a promising therapeutic intervention in treating a variety of disorders related to oxidative stress and inflammation. Nrf2, an upstream transcription factor modulating phase II enzyme activity, interacts with the antioxidant response element (ARE) in the nucleus to induce ARE-dependent gene expression. Under physiological conditions, Nrf2 is sequestered by binding to Kelch-like ECH-associated protein 1 (Keap1). Upon oxidative stress, Nrf2 parts from Keap1 and translocates into the nucleus to induce the expression of HO-1 [12]. Suppression of Nrf2 by the specific Nrf2 inhibitor, brusatol, has been reported to attenuate the Nrf2-mediated defense mechanism and weaken its antioxidant ability, indicating the importance of Nrf2 in antioxidant activity [13].

Previous studies have suggested that phosphatidylinositol 3-kinase (PI3K)/Akt is a key survival signaling pathway that enhances cellular defense, making it a potential treatment target not only by promoting cell survival, but also by modulating Nrf2 as an upstream signaling molecule [14–17]. Cross-talk between the PI3K/Akt and Nrf2 signaling pathways has been reported to govern the cellular defense system against inflammatory and oxidative damages [14]. LY294002, a specific PI3K/Akt inhibitor, could significantly attenuate the PI3K/Akt-mediated cell defense mechanism by suppressing phosphorylation of Akt and thus inhibiting activation of Nrf2/HO-1 expression [18]. Therefore, the PI3K/Akt pathway plays a vital role in the Nrf2-mediated antioxidant response, making it a potential target for medical intervention.

Many polyphenols that scavenge free radicals have been identified and proposed as therapeutic agents to counteract oxidative stress-induced diseases [19]. Recently, pomegranate extract, composed of abundant tannins, has been reported to exhibit antioxidant, anti-inflammation, and lipase inhibitory activities [20–23]. Punicalagin (2,3-hexahydroxydiphenoyl-gallagyl-D-glucose; PUN) is the major polyphenol isolated from pomegranate (*Punica granatum L.*), but few studies have been carried out focusing on its bioactivities. Our previous research has shown that PUN inhibits LPS-induced mitogen-activated protein kinases (MAPKs) and NF-*κ*B activation and thus suppresses overproduction of cytokines and inflammatory factors, including nitric oxide (NO), prostaglandin E2 (PGE2), interleukin- (IL-) 1*β*, IL-6, and tumor necrosis factor- (TNF-) *α* [24]. However, the antioxidant activity and mechanisms of PUN in macrophages remain unknown.

In this study, we investigated the PUN modulation of the Nrf2/HO-1 antioxidant signaling. Furthermore, we tried to uncover the molecular mechanism by which the PI3K/Akt pathway regulates the PUN-induced Nrf2/HO-1 activation and antioxidant activity. Moreover, we tried to reveal the underlying defense mechanism of PUN in LPS-stimulated macrophage oxidative stress.

## 2. Materials and Methods

### 2.1. Reagents

PUN (>98% HPLC purity) and brusatol were purchased from Tauto Biotech (Shanghai, China). LPS (*Escherichia coli* 055:B5) and insulin were purchased from Sigma-Aldrich Chemical (St. Louis, MO, USA). Fetal bovine serum (FBS), antibiotic-antimycotic, and TRIzol reagent were purchased from Gibco (Grand Island, NY, USA). Bicinchoninic acid (BCA) protein assay kit was purchased from Pierce (Rockford, IL, USA). Antibodies against GAPDH, Akt, p-Akt, Keap1, Nrf2 and HO-1, and LY294002 were purchased from Cell Signaling Technology (Danvers, MA, USA). The goat anti-mouse antibody was purchased from Li-cdr Odyssey (Lincoln, NE, USA). The probe, 2′,7′-dichlorodihydrofluorescein diacetate (DCFH_2_-DA), was purchased from Invitrogen (Carlsbad, CA, USA). The nitrate assay kit was purchased from Beyotime (Haimen, China).

### 2.2. Cell Line

RAW264.7 cells were purchased from the American Type Culture Collection (Rockville, MD, USA). Cells were cultured in DMEM medium supplemented with 10% FBS and antibiotics (100 U/mL penicillin and 100 U/mL streptomycin) at 37°C in a humidified incubator with 5% CO_2_.

### 2.3. NO Assay

The nitrite accumulated in the culture medium was measured as an indicator of NO production based on the Griess reaction. RAW264.7 cells, cultured in 96-well plates for 24 h, were treated with or without LPS (1 *μ*g/mL) in the presence of various doses of PUN (25, 50, or 100 *μ*M, 1 h prior to LPS treatment). After 24 h, culture supernatants were mixed with Griess reagent (equal volumes of 1% (w/v) sulfanilamide in 5% (v/v) phosphoric acid and 0.1% (w/v) naphthylethylenediamine-HCL) and incubated at room temperature for 10 min. Absorbance values were measured at 550 nm and NO concentration was calculated with reference to a standard curve of sodium nitrite.

### 2.4. Measurement of ROS Production

ROS production was detected by measuring intracellular ROS formation using the DCFH_2_-DA probe. Briefly, RAW264.7 cells, cultured in 24-well plates for 24 h before the experiment, were pretreated with PUN (100 *μ*M) for 1 h and then stimulated with LPS (1 *μ*g/mL) for 12 h to induce ROS production. Cells were washed twice with PBS and then incubated with DCFH_2_-DA probe (20 nM) for 15 min. Fluorescence staining was visualized using a fluorescence microscope (Olympus, IX71), and fluorescence assays were measured with a fluorescence microplate reader (Tecan, Sunrise) at excitation/emission 525/610 nm.

### 2.5. Western Blotting Analysis

RAW264.7 cells (1 × 10^6^) cultured in tissue culture flasks for 24 h were treated with the desired agents. Cells were then harvested on ice, washed twice with ice-cold PBS, and suspended in 500 *μ*L of lysis buffer supplemented with protease inhibitors (JianCheng, Nanjing, China). After 30 min incubation on ice, cell extracts were subjected to centrifugation (12,000 ×g) at 4°C for 15 min to get cell protein. Protein quantification was performed with a BCA protein assay kit. Proteins were separated by SDS-PAGE, electrotransferred to nitrocellulose membranes (Pierce), and then hybridized with the specific antibodies. Blots were normalized against GAPDH to correct for differences in protein loading. Densitometric values of immunoblot signals were obtained from three separate experiments using Image J (National Institutes of Health, Bethesda, MD, USA).

### 2.6. Quantitative RT-PCR Analysis

RAW264.7 cells were preincubated in 6-well plates (1 × 10^6^) and pretreated with PUN (25, 50, or 100 *μ*M) 1 h prior to 1 *μ*g/mL LPS treatment for 12 h in a 37°C and 5% CO_2_ incubator. Total RNA was extracted using TRIzol reagent. The concentration and integrity of the RNA were measured at a 260/280 nm ratio. Quantitative PCR analysis was carried out using the DNA Engine Mx3000P fluorescence detection system (Agilent, Santa Clara, CA, USA) against a double-stranded DNA-specific fluorescent dye (Stratagene, La Jolla, CA, USA) according to optimized PCR protocols. *β-Actin* was amplified in parallel with the target genes and used as a normalization control. The PCR conditions were as follows: 95°C for 3 min, followed by 40 cycles of 95°C for 10 s, 60°C for 20 s, and 72°C for 60 s. Expression levels were determined using the relative threshold cycle (CT) method as described by the manufacturer (Stratagene). The PCR reaction system (25 *μ*L in total) contained 12.5 *μ*L of SYBR Green PCR mix (Stratagene), 0.375 *μ*L of reference dye, 1 *μ*L of each primer (both 10 *μ*M), 1 *μ*L of cDNA template, and 9.125 *μ*L of DEPC-treated water. Quantitative real-time RT-PCR was performed using the following primers: *β-actin*, F: 5′-CCCATCTATGAGGGTTACGC-3′, R: 5′-TTTAATGTCACGCACGATTT C-3′;* SOD1*, F: 5′-CCACGTCCATCAGTATGGGG-3′, R: 5′-CGTCCTTTCCAGCAGTCACA-3′;* SOD2*, F: 5′-GTGTCTGTGGGAGTCCAAGG-3′, R: 5′-CCCCAGTCATAGTGCTGCAA-3′;* HO-1*, F: 5′-CACGCATATACCCGCTACCT-3′, R: 5′-CCAGAGTGTTCATTCGAGCA-3′;* Nrf2*, F: 5′-AACAGAACGGCCCTAAAGCA-3′, R: 5′-TGGGATTCACGCATAGGAGC-3′.

## 3. Statistical Analysis

The results were expressed as mean ± SEM and differences between mean values of normally distributed data were assessed by the one-way analysis of variance (ANOVA) followed by Duncan's test for multiple comparisons. *P* value of 0.05 or 0.01 was considered statistically significant.

## 4. Results

### 4.1. PUN Increases Nrf2/HO-1 Expression in RAW264.7 Cells

**Figure 1:**
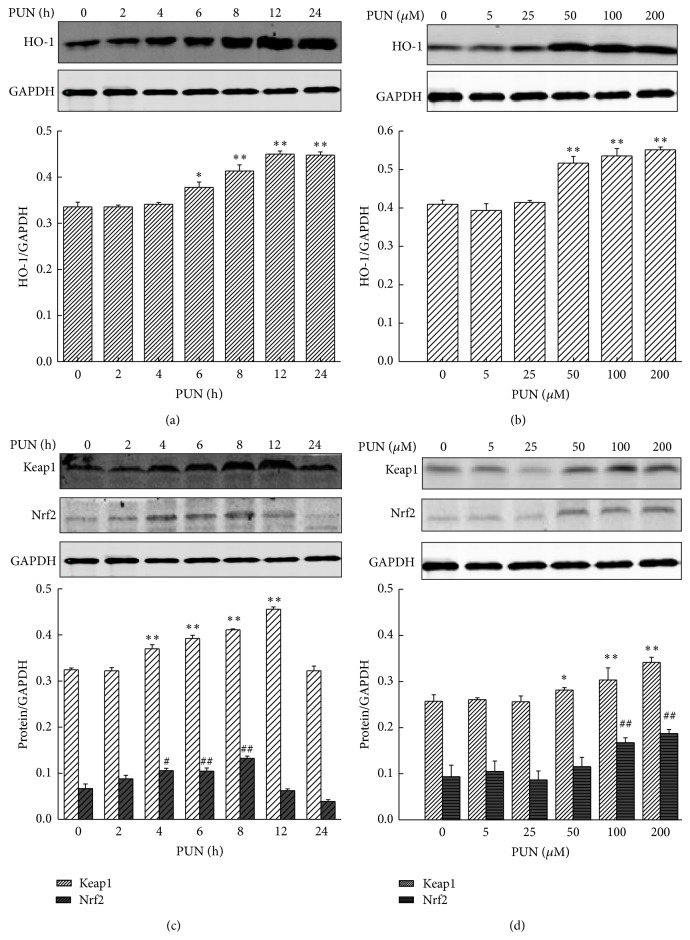
PUN increases Nrf2/HO-1 expression in RAW264.7 cells in time-dependent manner and dose-dependent manner. Cells were incubated at 37°C in a humidified incubator with 5% CO_2_. (a) RAW264.7 cells were treated with 100 *μ*M PUN for indicated durations (0, 2, 4, 6, 8, 12, and 24 h). The HO-1 protein expression was analyzed by Western blotting. (b) RAW264.7 cells were treated for 8 h with PUN at the indicated concerntrations (0, 5, 25, 50, 100, and 200 *μ*M). The HO-1 protein expression was analyzed by Western blotting. (c) RAW264.7 cells were treated with 100 *μ*M PUN for indicated durations (0, 2, 4, 6, 8, 12, and 24 h). The Nrf2 and Keap1 protein expression were analyzed by Western blotting. (d) RAW264.7 cells were treated for 8 h with PUN at the indicated concerntrations (0, 5, 25, 50, 100, and 200 *μ*M). The Nrf2 and Keap1 protein expression were analyzed by Western blotting. Data represent the mean ± SEM of three independent experiments and differences between mean values were assessed by one-way ANOVA. ^∗^ *P* < 0.05, ^∗∗^ *P* < 0.01, ^#^ *P* < 0.05, and ^##^ *P* < 0.01 indicate significant differences compared with the control group of indicated proteins, respectively.

**Figure 2:**
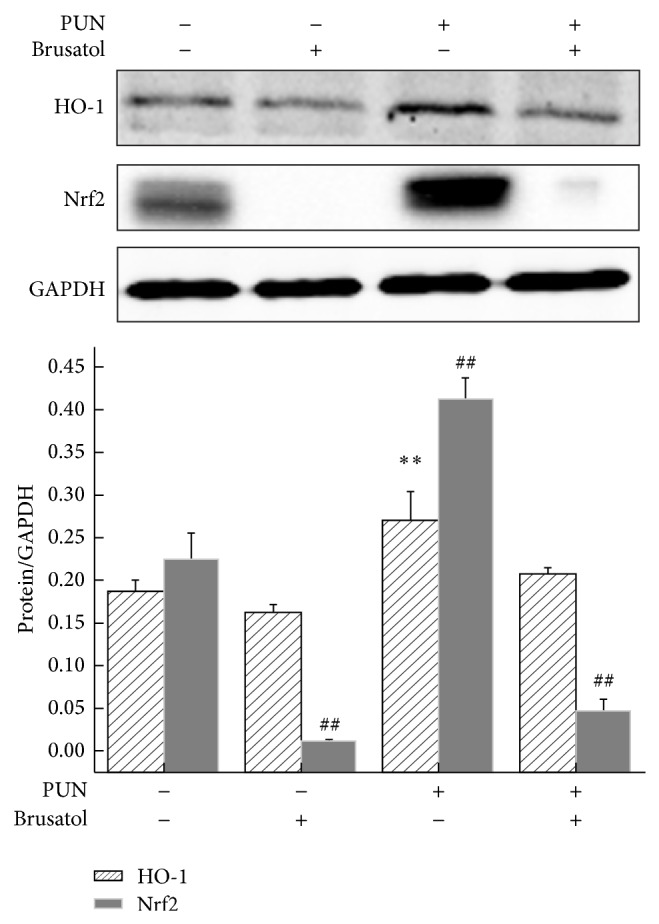
Inhibition of Nrf2 suppresses PUN induced HO-1 protein expression in RAW264.7 cells. Cells were treated for 8 h with 100 *μ*M PUN in the presence or absence of 20 nM brusatol at 37°C in a humidified incubator with 5% CO_2_. The HO-1 and Nrf2 protein expression were analyzed by Western blotting. Data represent the mean ± SEM of three independent experiments and differences between mean values were assessed by one-way ANOVA. ^∗∗^ *P* < 0.01 and ^##^ *P* < 0.01 indicate significant differences compared with the control group.

HO-1 expression was measured to determine whether PUN exhibits potential antioxidant activity by upregulating the intracellular phase II enzyme, HO-1, in RAW264.7 cells. Western blot analysis was performed to detect the expression of HO-1 induced by different concentrations of PUN and as a function of time. The results showed that PUN started to significantly increase HO-1 protein levels from 6 h in a time-dependent manner (Figure 1(a)). Furthermore, PUN enhanced HO-1 protein levels at doses of 50 to 200 *μ*M in a dose-dependent manner (Figure 1(b)). To further characterize the molecular mechanism of PUN activity, Nrf2, a vital upstream signaling mediator of HO-1, was examined by Western blot analysis. The data demonstrated that 100 *μ*M PUN treatment significantly increased Nrf2 expression from 4 h to 8 h in a time-dependent manner (Figure 1(c)). Also, an 8 h treatment with 50 to 200 *μ*M PUN increased Nrf2 expression in a dose-dependent manner (Figure 1(d)). Keap1 functions as a suppressor of Nrf2 by retaining it in the cytoplasm and enhancing its proteasomal degradation via ubiquitination. Keap1 deficiency leads to Nrf2 isolation and translocation into the nucleus. However, in our study PUN treatment also increased Keap1 accumulation from 4 h to 12 h (100 *μ*M) and at doses of 50 to 200 *μ*M (8 h) (Figures 1(c) and 1(d)). RT-PCR results also showed that PUN could significantly increase the Nrf2 and HO-1 mRNA expression since 4 h treatment. However, we found that 24 h treatment still showed an enhancement in Nrf2 mRNA expression but not in Nrf2 protein expression, indicating that the inhibition effect of PUN on Keap1-Nrf2 could not last more than 24 h (Figure 1S available online at http://dx.doi.org/10.1155/2015/380218). Thus, we speculated that PUN increased Nrf2 accumulation and activation by inhibiting Keap1 ubiquitination of Nrf2 and/or the physical interaction, thereby preventing Nrf2 from being degraded. In the 12 h PUN treatment group, the Keap1 level significantly increased, while the Nrf2 level notably decreased, suggesting that the inhibitory effect of PUN (100 *μ*M) on the Keap1-Nrf2 ubiquitination sustained no longer than 12 h, as Keap1 rapidly accumulated and degraded Nrf2 protein, keeping Nrf2 at a low concentration. Brusatol is a specific Nrf2 inhibitor, suppressing its translocation and preventing it from inducing ARE-mediated antioxidant genes, including* HO-1*. We used brusatol to block Nrf2 activity and found that the PUN-induced HO-1 accumulation was significantly attenuated (Figure 2), suggesting that PUN induced HO-1 upregulation via Nrf2-mediated signaling.

### 4.2. PI3K/Akt Regulates PUN-Induced HO-1 Expression in RAW264.7 Cells

**Figure 3:**
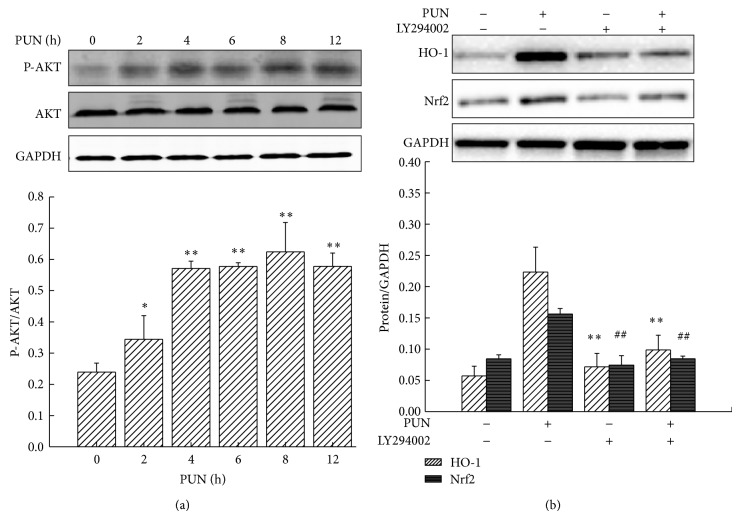
PUN induced HO-1 protein expression via PI3K/Akt pathway. Cells were incubated at 37°C in a humidified incubator with 5% CO_2_. (a) RAW264.7 cells were treated with 100 *μ*M PUN for indicated durations (0, 2, 4, 6, 8, and 12 h). The Akt protein and Akt phosphorylation level were analyzed by Western blotting. (b) RAW264.7 cells were treated for 8 h with 100 *μ*M PUN in the presence or absence of 20 *μ*M LY294002. The HO-1 and Nrf2 proteins expression were analyzed by Western blotting. Data represent the mean ± SEM of three independent experiments and differences between mean values were assessed by one-way ANOVA. ^∗∗^ *P* < 0.01 and ^##^ *P* < 0.01 indicate significant differences compared with the PUN-treated group of indicated proteins, respectively.

**Figure 4:**
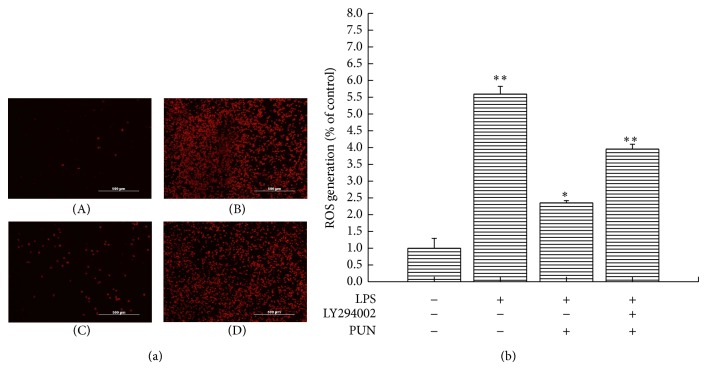
PI3K/Akt pathway regulates property of PUN on ROS scavenging. (a) ROS detection was performed using a fluorescence macroscopy. (A) RAW264.7 cells were cultured in DMEM for 12 h and then incubated with probe DCFH2DA for 15 min. (B) RAW264.7 cells were treated with 1 *μ*g/mL LPS for 12 h and then incubated with probe DCFH2DA for 15 min. (C) RAW264.7 cells were pretreated with 100 *μ*M PUN for 1 h and treated with 1 *μ*g/mL LPS for 12 h and then incubated with probe DCFH2DA for 15 min. (D) RAW264.7 cells were pretreated with 100 *μ*M PUN and 20 *μ*M LY294002 for 1 h and treated with 1 *μ*g/mL LPS for 12 h and then incubated with probe DCFH2DA for 15 min. (b) ROS production was measured by a fluorescence microplate reader. RAW264.7 cells were pretreated for 1 h with 100 *μ*M PUN in the presence or absence of 20 *μ*M LY294002 before treatment with 1 *μ*g/mL LPS for 12 h and then incubated with probe DCFH2DA for 15 min. Data represent the mean ± SEM of three independent experiments and differences between mean values were assessed by one-way ANOVA. ^∗^ *P* < 0.05 and ^∗∗^ *P* < 0.01 indicate significant differences compared with the control group.

Recent studies have demonstrated that the PI3K/Akt pathway acts as an important upstream regulator of HO-1 expression [25]; thus we investigated whether the PI3K/Akt pathway also plays a central role in the PUN-induced HO-1 expression. Western blot analysis showed that PUN treatment notably enhanced Akt phosphorylation after 2 h of treatment in a time-dependent manner, but not the total Akt protein level, suggesting that the PUN-induced HO-1 expression may be associated with the PI3K/Akt signaling pathway (Figure 3(a)). To further reveal the mechanism, we used LY294002, a specific PI3K/Akt inhibitor. The results showed that PUN treatment significantly increased HO-1 expression; however, inhibiting Akt phosphorylation by LY294002 significantly attenuated the PUN-induced HO-1 activation (Figure 3(b)). Therefore, we concluded that PI3K/Akt is essential for the PUN antioxidant activity by regulating PUN-induced HO-1 expression. LPS treatment triggers ROS generation in macrophages, so we used LPS to determine whether PI3K/Akt signaling regulates the ROS scavenging property of PUN. As expected, both fluorescence staining and assay values demonstrated that the PI3K/Akt pathway regulates the ability of PUN to scavenge ROS (Figure 4), which is in accordance with its effect on HO-1 expression.

### 4.3. Nrf2 Is Essential for PI3K/Akt-Mediated HO-1 Expression in PUN-Treated RAW264.7 Cells

**Figure 5:**
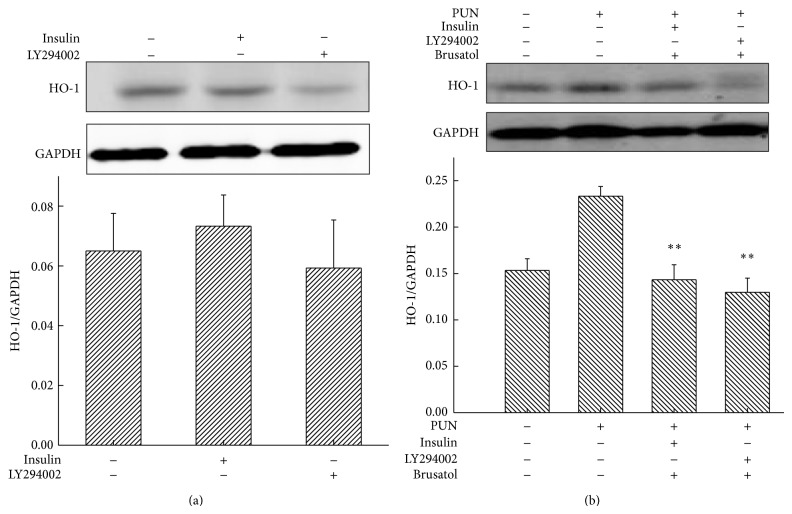
Blocking Nrf2 suppresses modulatory effect of PI3K/Akt pathway on PUN-induced HO-1 protein expression. RAW264.7 cells were treated for 8 h with 100 *μ*M PUN in the presence or absence of indicated inhibitor (LY294002, 20 *μ*M; brusatol, 10 nM) and inducer (insulin, 10 *μ*M) at 37°C in a humidified incubator with 5% CO_2_. The HO-1 protein expression was analyzed by Western blotting. Data represent the mean ± SEM of three independent experiments and differences between mean values were assessed by one-way ANOVA. ^∗∗^ *P* < 0.01 indicates significant differences compared with the PUN-treated group.

The results showed that LY294002 inhibited the PUN-induced activation of Nrf2 (Figure 3(b)), indicating that Nrf2 may be a vital molecule in the PI3K/Akt-mediated HO-1 expression in PUN-treated cells. To further understand this mechanism, brusatol was used to suppress the Nrf2-mediated signaling pathway in the presence of PI3K/Akt mediators. We found that upregulation (insulin) or downregulation (LY294002) of PI3K/Akt in presence of PUN did not show a significant modulation effect on HO-1 expression when Nrf2 was blocked (Figure 5), indicating that Nrf2 plays a central role in the PI3K/Akt-regulated HO-1 expression in PUN-treated cells. Hence, we concluded that Nrf2 is downstream PI3K/Akt, but upstream HO-1 in the PUN-regulated antioxidant signaling pathway.

### 4.4. PUN Inhibits LPS-Induced Oxidative Stress in RAW264.7 Cells

**Figure 6:**
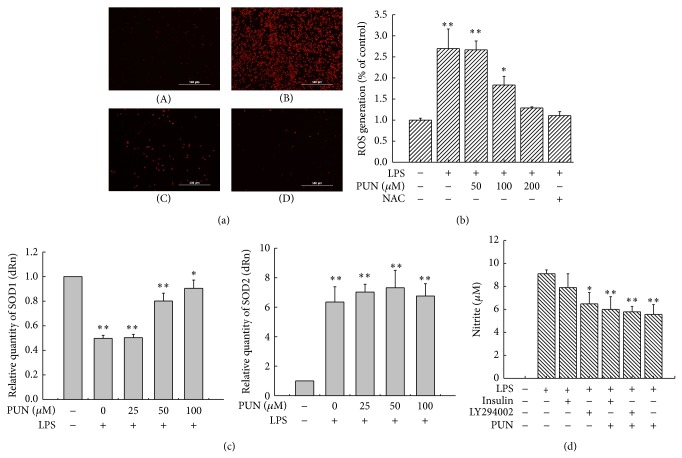
PUN inhibits LPS-induced oxidative stress. (a) PUN inhibits LPS-induced ROS generation. (A) RAW264.7 cells were cultured in DMEM for 12 h and then incubated with probe DCFH2DA for 15 min. (B) RAW264.7 cells were treated with 1 *μ*g/mL LPS for 12 h and then incubated with probe DCFH2DA for 15 min. (C) RAW264.7 cells were pretreated with 100 *μ*M PUN for 1 h before treatment with 1 *μ*g/mL LPS for 12 h and then incubated with probe DCFH2DA for 15 min. (D) RAW264.7 cells were pretreated with 50 *μ*M NAC for 1 h before treatment with 1 *μ*g/mL LPS for 12 h and then incubated with probe DCFH2DA for 15 min. (b) ROS detection was performed by a fluorescence microplate reader. RAW264.7 cells were pretreated for 1 h with PUN at indicated doses (0, 50, 100, and 200 *μ*M) in the presence or absence of 100 *μ*M NAC and before treatment with 1 *μ*g/mL LPS for 12 h and then incubated with probe DCFH2DA for 15 min. (c) PUN mediated SOD1 and SOD2 mRNA expression in LPS-treated RAW264.7 cells. Cells were pretreated with PUN (25, 50, and 100 *μ*M) and then treated to 1 *μ*g/mL LPS for 12 h; SOD1 and SOD2 mRNA expression were detected using RT-PCR. (d) PUN inhibits LPS-induced NO overproduction; RAW264.7 cells were pretreated for 1 h with or without 100 *μ*M PUN in the presence or absence of indicated inhibitor (LY294002, 20 *μ*M) and inducer (insulin, 10 *μ*M) before treatment with 1 *μ*g/mL LPS for 12 h. NO production in the supernatant was measured using Griess reaction. Data represent the mean ± SEM of three independent experiments and differences between mean values were assessed by one-way ANOVA. ^∗^ *P* < 0.05, ^∗∗^ *P* < 0.01, ^△^ *P* < 0.05, ^△△^ *P* < 0.01, ^#^ *P* < 0.05, and ^##^ *P* < 0.01 indicate significant differences compared with the control group of indicated proteins, respectively.

When stimulated with LPS, the intracellular ROS level in macrophages increased rapidly, causing oxidative stress. We then tried to evaluate the antioxidant activity of PUN by examining ROS generation and* SOD1*/*SOD2* mRNA expression. LPS stimulation significantly increased the ROS level in macrophages, but pretreatment with PUN notably prevented the LPS-induced ROS generation. As a well-established ROS scavenger, N-acetyl cysteine (NAC) treatment also showed a significant inhibitory effect on the LPS-induced ROS overproduction. The inhibitory effect of 200 *μ*M PUN and 50 *μ*M NAC treatment was comparable (Figures 6(a) and 6(b)). RT-PCR analysis showed that LPS significantly reduced* SOD1* mRNA expression and enhanced* SOD2* mRNA expression. PUN pretreatment significantly reversed the reduction in* SOD1* mRNA caused by LPS but showed no effect on* SOD2* mRNA expression (Figure 6(c)). NO is another important free radical molecule and proinflammatory factor in LPS-stimulated macrophages. Because we revealed that the PI3K/Akt pathway is essential for PUN to scavenge intracellular ROS, we examined the role of PI3K/Akt in the reduction of NO production in PUN-treated cells. The results showed that PUN pretreatment notably decreased the LPS-induced NO overexpression in macrophages; however, upregulation or downregulation of PI3K/Akt did not affect the PUN-induced reduction in NO, suggesting that PUN inhibited the LPS-induced NO expression via another signaling pathway Figure 6(d).

## 5. Discussion

Pomegranate extracts have been reported to have many beneficial health effects, exhibiting antioxidant, anti-inflammation, antiproliferative, and DNA repair activities, which are generally attributed to the high polyphenol content [26–28]. The pomegranate husk is rich in polyphenols such as punicalagin (PUN), punicalin, gallagic acid, ellagic acid, and EA-glycosides [29]. PUN is a hydrolysable polyphenol in which gallagic acids are linked to a sugar moiety with a molecular weight of 1084 [30]. Previous studies have shown that PUN composes 85% of the total pomegranate tannins and accounts for more than 50% of the antioxidant activity of pomegranate juice [29, 31]. An increasing number of studies indicated that the bioactivities of pomegranate extracts are associated with PUN [32, 33]. Our recent study has demonstrates that PUN inhibits LPS-induced inflammatory factors and cytokine overexpression, including NO, PGE2, IL-1*β*, IL-6, and TNF-*α*, via suppression of toll-like receptor 4-mediated MAPKs and NF-*κ*B activation in macrophages, which may contribute to the inhibition effect of pomegranate on inflammation [24]. Oxidative stress caused by LPS or other stimuli may trigger the activation of macrophages, leading to an excessive inflammatory process. However, as a polyphenol possessing most of the pomegranate's antioxidant activity, only limited studies have been carried out on the antioxidant property of PUN and its underlying mechanism in macrophages.

HO-1 can be stimulated by a variety of factors, including heme, hyperoxia, and ROS in most cell types, and studies have revealed that upregulation of HO-1 contributes to the cellular defense mechanism in response to stimuli [34]. LPS challenge rapidly increases the ROS level in macrophages, not only directly damaging DNA, but also inducing overproduction of inflammatory factors as well as cytokines, which may lead to severe tissue injury [35]. Macrophages are essential for recognizing and eliminating microbial pathogens, and thus the survival of macrophages may directly contribute to the host defense system. Furthermore, counteracting the overproduction of ROS is crucial for inhibiting excessive inflammation. Therefore, HO-1, which is important for protecting macrophages from ROS, has recently become a popular target for antioxidant medicine development [36–38]. The present study showed that PUN treatment markedly increased HO-1 protein level in macrophages in a time- and dose-dependent manner, indicating that PUN exhibited antioxidant activity by upregulating HO-1 expression.

Nrf2 is known as an upstream mediator of ARE-dependent phase II enzyme expression, including HO-1 [39–41]. Under quiescent conditions, Nrf2 is anchored in the inactive form to the cytoplasm through binding to Keap1, which in turn facilitates the ubiquitination and subsequent proteolysis of Nrf2 protein. The sequestration and further degradation of Nrf2 are mechanisms for the repressive effect of Keap1 and Nrf2. To investigate whether PUN upregulates HO-1 via enhancing Nrf2 accumulation and activation, protein was extracted and the Nrf2 protein level was examined by Western blot analysis. The results showed that PUN treatment significantly increased Nrf2 protein level in the nucleus from 4 h to 8 h and in a dose range of 50 *μ*M to 200 *μ*M, suggesting that PUN may regulate HO-1 expression by mediating Nrf2 signaling. We used brusatol, a specific Nrf2 inhibitor, to determine whether blocking Nrf2-mediated signaling attenuates PUN-induced HO-1 expression. As expected, we found that inhibition of Nrf2 markedly suppressed PUN-induced HO-1 expression. Therefore, we concluded that PUN exhibited antioxidant activity via upregulating Nrf2-mediated HO-1 expression. However, as shown in Figures 1(c) and 1(d), the protein level of Keap1 also increased by PUN treatment. As a CUL3-RBX1-dependent E3 ubiquitin ligase, Keap1 degrades Nrf2 by directly interacting with it and conjugating ubiquitin onto the N-terminal Neh2 domain of Nrf2 [42]. Thus, we hypothesized that PUN may also affect the interaction between Keap1 and Nrf2. Further research will be carried out in the future to examine this mechanism.

The PI3K/Akt signaling pathway is also involved in regulating HO-1 expression [43]. Therefore, we investigated the possibility that PI3K/Akt mediates PUN-induced HO-1 expression. As shown in Figure 3(a), PUN treatment notably increased the phosphorylation level of Akt protein, while no significant changes were found in the total Akt protein level, suggesting that enhancement of Akt protein phosphorylation may contribute to the PUN-induced HO-1 expression. To further identify the role of the PI3K/Akt pathway, LY294002, a specific PI3K/Akt inhibitor, was used to treat macrophages together with PUN. The results showed that inhibition of the PI3K/Akt signaling pathway markedly blocked HO-1 expression in the presence of PUN. Furthermore, we investigated whether PI3K/Akt plays an important role in ROS scavenging by PUN, in accordance with its regulation of HO-1 expression. Fluorescent probes showed that blocking PI3K/Akt signaling by LY294002 attenuated the ROS scavenging capability of PUN in LPS-induced oxidative stress in macrophages, and this may be related to the downregulation of HO-1 expression.

Because the PI3K/Akt and Nrf2 pathways are both implicated in the transcriptional regulation of the antioxidant enzyme HO-1 [44], we tried to reveal their underlying mechanisms in the PUN-induced HO-1 protein expression. The data demonstrated that inhibition of PI3K/Akt significantly suppressed Nrf2 protein expression induced by PUN. Then, we examined whether blocking Nrf2 inhibits the PI3K/Akt-mediated HO-1 expression in the presence of PUN. The results demonstrated that inhibition of Nrf2 suppressed the upregulation of HO-1 by PUN treatment in macrophages; however, neither LY294002 nor insulin had a significant effect when the Nrf2 signaling was blocked. Therefore, we concluded that the PI3K/Akt signaling pathway played a vital role in PUN-induced HO-1 expression by regulating Nrf2.

ROS overproduction plays a central role in LPS-induced macrophage activation, leading to an excessive inflammatory process. In this study, we assessed the inhibitory effect of PUN on ROS production. Compared with the control group, LPS treatment significantly increased ROS generation. However, pretreatment with PUN dramatically decreased ROS production. NAC, a well-established ROS scavenger, was used to treat macrophages as a positive control, and 50 *μ*M NAC significantly decreased the LPS-induced ROS overproduction, to a similar extent as 200 *μ*M PUN treatment. SOD, which catalyzes the dismutation of superoxide radical (O_2_^−^) to hydrogen peroxide (H_2_O_2_) and oxygen (O_2_), is another kind of phase II enzyme mediated by Nrf2 that serves as a defense mechanism against oxidative damage [45]. SOD1 (Cu/Zn-SOD) locates primarily in the cytoplasm, and SOD2 (Mn-SOD), a structurally distinct protein, locates in the mitochondria [46]. Our results demonstrated that a 12 h LPS challenge significantly reduced the* SOD1* mRNA expression and increased* SOD2* mRNA expression in macrophages. However, PUN pretreatment only notably reversed the* SOD1* mRNA expression, with no effect on* SOD2* mRNA expression. NO is another important molecule regulated by the Nrf2-mediated pathway, whose excessive generation has been shown to result in oxidative stress and inflammation during an LPS challenge [37, 47]. Previous studies have indicated that the PI3K/Akt pathway is a potential target of medicines that exhibit an NO inhibition effect in LPS-induced macrophages [48, 49]. Hence, we investigated whether PI3K/Akt signaling also modulates the inhibition property of PUN on NO generation in macrophages. PUN treatment significantly weakened the LPS-induced NO overproduction. However, comparing LY294002 treatment with insulin treatment, we concluded that upregulation or downregulation of PI3K/Akt showed no significant changes in decreasing the LPS-induced NO expression in the presence of PUN, indicating that PI3K/Akt signaling was not involved in the inhibitory effect of PUN on NO expression in macrophages. Our previous study has revealed that PUN inhibits LPS-induced NO overproduction by suppressing MAPKs/NF-*κ*B activation. In that study, we inferred that PUN inhibited NO expression through MAPKs signaling, but not through PI3K/Akt signaling. Thus, we concluded that PUN exhibited antioxidant properties, including scavenging ROS, potentiating* SOD1* expression, and inhibiting NO production.

Taken together, these results show that PUN enhances HO-1 expression by upregulating the Nrf2-mediated pathway in macrophages and that the PI3K/Akt pathway plays a central role in this mechanism. Our data also demonstrated that PUN exhibits considerable antioxidant properties in LPS-stimulated macrophages by inhibiting ROS generation and NO overproduction and by enhancing the* SOD1* mRNA expression. These findings provide new perspectives for novel therapeutic approaches using antioxidant medicines and compounds against oxidative stress and excessive inflammatory diseases including tissue damage, sepsis, and endotoxemic shock.

## Supplementary Material

PUN showed a significantly enhancement the mRNA expression of HO-1 and Nrf2 since 4h treatment in RAW264.7 cells.
