# Supplementary material for: Punicalagin Induces Nrf2/HO-1 Expression via Upregulation of PI3K/AKT Pathway and Inhibits LPS-Induced Oxidative Stress in RAW264.7 Macrophages
Source: Mediators Inflamm. 2015 Apr 19;2015:380218. doi: 10.1155/2015/380218 (PMC4417599; doi:10.1155/2015/380218)
Supplement: Supplementary file 1 — PUN showed a significantly enhancement the mRNA expression of HO-1 and Nrf2 since 4h treatment in RAW264.7 cells. [file 380218.f1.docx]

**Figure 1 S**





**Figure 1 S.** PUN effect on *Nrf2* and *HO-1* mRNA expression in RAW264.7 cells. Cells were pretreated with 100 μM PUN for indicated time course. *Nrf2* and *HO-1* mRNA expression was detected using RT-PCR. Data represent the mean ± S.E.M of three independent experiments and differences between mean values were assessed by one-way ANOVA. *p＜0.05, **p＜0.01 ^#^p＜0.05 and ^##^p＜0.01 indicate significant differences compared with the control group of indicated proteins, respectively.
